# Pain relieving effects of Botox injection in the hip joint following a periacetabular osteotomy

**DOI:** 10.1093/jhps/hnaf019

**Published:** 2025-04-14

**Authors:** Niels Bang, Bjarne Mygind-Klavsen, Bent Lund, Casper Foldager, Stig Storgaard Jacobsen

**Affiliations:** Department of Radiology, Regionshospitalet, Falkevej 1, Silkeborg 8600, Denmark; Department of Orthopedic, Aarhus University Hospital, Palle Juul-Jensens Boulevard 99, Aarhus 8200, Denmark; Department of Orthopedic, Regionshospitalet Horsens, Sundvej 30, Horsens 8700, Denmark; Sana Hypoxi, Frisenborgvej 3L, 1.sal, 8240 Risskov, Risskov 8200, Denmark; Department of Orthopedic, Aarhus University Hospital, Palle Juul-Jensens Boulevard 99, Aarhus 8200, Denmark

## Abstract

Patients with symptomatic hip dysplasia may undergo periacetabular osteotomy (PAO) and 10% of these patients have pain >6 months after the operation. An intra-articular lidocaine injection is used to determine if the pain comes from labral pathology in the hip joint or structures around the hip. To allow the patient a longer period of time to test the hip, we wanted to test if an intra-articular injection of Botox combined with local anaesthetic could reduce pain and allow the patient to test the hip. Eleven patients who received a PAO and suffered from persistent pain at least 6 months postoperative had an intra-articular joint injection with 100IE Botox, 3 ml NaCl water and 3 ml lidocaine. Hip pain on the day of injection and after 6 weeks was evaluated using the Visual Analog Scale score, International Hip Outcome Tool (iHOT-12), and Hip and Groin Outcome Score (HAGOS). Botox in the hip joint reduced the pain level and the iHOT-12 score, with a statistically significant improvement 6 weeks after injection. Hip joint injection of a controlled dosage of 100 IU diluted in 3 ml NaCl solution mixed with lidocaine had no side effects in this study with a limited group of patients. Botox has a promising pain-reducing effect on the hip joint in the majority of patients comparable with findings in knee and shoulder joints. Hip joint injection of a controlled dosage of 100 IU, 3 ml NaCl solution mixed with lidocaine had no side effects in this study with a limited group of patients.

## Introduction

Botulinum toxin is believed to temporarily inhibit nerve transmission in pain transmitters and autonomic nerves and reduce the release of substance P and other inflammatory mediators [1]. Approved for the treatment of musculoskeletal and autonomic disorders, such as hyperhidrosis, and chronic pain conditions including myofascial pain syndrome, migraines, and tension headaches, Botox also influences sensory feedback by reducing signals in afferent 1a fibres [2]. Sensory effect of the transient receptor potential vanilloid 1 plays an important role in the perception of peripheral thermal and inflammatory pain [3]. This suggests that Botox not only provides direct pain relief but also may have a broader desensitizing effect, offering an enhanced therapeutic benefit in treating hip pain and associated symptoms. Due to this, we hypothesized that intra-articular Botox could inhibit pain transduction from the hip and desensitize nerves, providing significant and sustained pain reduction with minimal side effects.

Young patients with symptomatic developmental hip dysplasia may undergo periacetabular osteotomy (PAO) [4]. It is common for these patients to have labral and chondral pathologies, and some patients continue to report groin pain postoperatively [5]. Pain in the nonarthritic hip joint in particular following a PAO can often be related to pathological findings in the labrum and adjacent synovium. The pain is often felt in the groin and deep in the buttock and is usually accompanied by restricted mobility and impaired function. A standard procedure to localize the pain is local anaesthesia injected in the hip joint to determine if the pain can be relieved and at the same time clarify whether the pain originates from the joint itself or from structures around the hip joint [6].

Patients are typically diagnosed using intra-articular injections of 8 ml of 10 mg/ml lidocaine, a local anaesthetic known to cause no side effects [7]. However, if inflammation is suspected, e.g. if there is significant thickening of the synovia in the hip joint—>1 mm compared to the opposite side or synovia thicker than 7 mm (WA Schmidt Standard reference values for musculoskeletal ultrasonography)—40–80 mg of corticosteroid is frequently added to the anaesthetic to prolong the pain relief [8]. Injection with at least 8 ml 10 mg/ml lidocaine and corticosteroid allows the patient 3 h of diagnostic effect from the local anaesthetic were any pain from the hip—during this time must be suspected to come from structures around the hip. At the same time, the injection allows the corticosteroid to give a longer-lasting effect after the initial evaluation of the local anaesthetic effect that lasts ∼3 h. Informing the patient about evaluating the pain during the first 3 h post-injection separated from the following corticosteroid effect is important and can be helped with a score sheet for the registration of pain during the first 3 h after the medicine injection and the following effect. Although effective, corticosteroids can have several undesirable side effects, including flushing, menstrual disorders, and disturbances in blood glucose levels, potentially elevated risk of thrombosis, osteoporosis, and fractures [9].

In response to these challenges, the introduction of Botox offers a promising alternative [10]. By providing longer-lasting pain relief, Botox allows for a more thorough evaluation period post-treatment, during which patients and healthcare providers can assess the effectiveness of the intervention. This extended relief also enables patients to test their mobility in daily activities, potentially leading to better-informed decisions about further treatment options. Botox has previously been tested for knee pain with a similar dose, where pain reduction between 2 and 4.2 points on the Visual Analog Scale (VAS) scale (0 is no pain and 10 is the worst possible pain) was found in the period 8 weeks to 3 months after injection [11–13].

This pilot study aims to explore the effect of intra-articular injections combining Botox with lidocaine in the hip joint as a diagnostic tool for evaluating hip joint pain in nonarthritic hip joints with a high suspicion of labral pathology. Additionally, the study aims to assess the persistence of pain relief up to 6 weeks’ post-injection and to identify any side effects. Finally, we also wanted to evaluate the safety by registering the adverse effects following the injection. The perspective of this innovative approach is intended to provide longer-lasting pain relief compared to traditional methods using only local anaesthetic, potentially improving diagnostic accuracy and safety. By extending the duration of pain relief, patients are afforded more time to assess reductions or even the elimination of pain, enabling them and their healthcare providers to make more informed decisions regarding further treatment. Furthermore, this preliminary investigation will inspire further research into other clinical indications where Botox may have a beneficial, long-lasting pain-relieving effect.

## Material and methods

### Design

This study constitutes a pilot study in a prospective cohort of 10 patients.

### Population

As an initial investigation, we focused on patients experiencing persistent hip pain 6 months after undergoing PAO. These patients have clinical signs of intra-articular pathology and no radiological signs of arthrosis.

### Inclusion criteria

- Patients with unresolved hip pain at least 6 months’ post-operative after PAO and no visible osteoarthritis on X-ray. Eligibility is confirmed during a consultation with a hip surgeon.
- Patients must be 18 years or older and have signed informed consent.
- Female participants of childbearing potential must test negative for pregnancy and are advised to use effective contraception for 3 months’ post-injection.
- Patients on regular pain medication are included, with any changes in their medication documented.

### Exclusion criteria

- Patients with neuromuscular diseases, or allergies to Botox, lidocaine, or isotonic NaCl.
- Patients physically or mentally unfit for follow-ups or with a BMI over 35.
- Patients who have received intra-articular corticosteroids or Botox injections in the affected joint within the last 3 months.
- Breastfeeding patients and blood donors who cannot abstain from donating blood for 4 months’ post-treatment.

### Treatments

**Figure 1.**
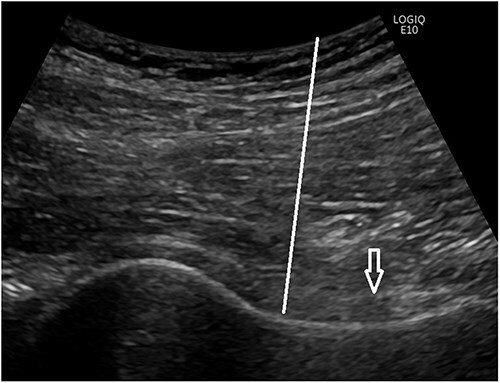
Ultrasound picture of the hip joint. The capsule of the hip joint is marked by an open arrow and the puncture line for Botox injection is marked with a line.

**Figure 2.**
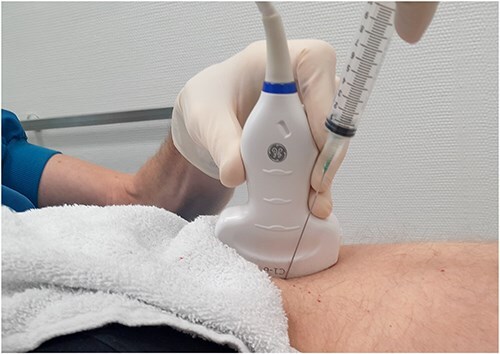
Illustration of how injection in the hip joint is made simple by free-hand injection and puncture on the side of the transducer.

The Botox injections were administered under ultrasound guidance using a solution of 100 IU Botox type A toxin mixed with lidocaine, ensuring precision, and minimizing discomfort. The medicine was injected under the capsule of the hip joint about 1 cm distal to the femoral head–neck junction (Figs 1 and 2). Medicine was injected into the joint under the capsule and care was taken so the Botox was not caught in the capsule. Where a corticosteroid injection in the capsule works anyway we suspect that the Botox injection only works when injected into the joint. During the follow-up period, patients were encouraged to report any side effects, with particular attention to any serious, unexpected, or late effects. All reported side effects were documented alongside any symptomatic treatments used. The effect of Botox is believed to begin within 1–3 weeks and can last from 3 to 6 months. For injections into the hip joint, we used 100 IU of Botox type A toxin from Allergan, dissolved in 3 ml of sodium chloride 9 mg/ml from Fresenius Kabi, and mixed with 3 ml of Xylocaine (Lidocaine) 10 mg/ml from Astra Zeneca. A precipitation test was conducted at Glostrup Pharmacy using the same doses to ensure that no precipitation occurred. The test confirmed that no precipitation was observed. Therefore, this combination of three registered medicines, repurposed for a new indication, constitutes a new investigational medicinal product.

### Outcome measures

Patients underwent movement tests Flexion, Adduction, Internal Rotation (FADIR) and pain assessments on Day 1 and were evaluated using standardized questionnaires (iHOT-12 [14]) and HAGOS [15]) and pain scales (Numeric Rating Scale (NRS)/VAS [16]) immediately before and after the Botox injections and during follow-up visits at 1, 2, and 6 weeks. These assessments (iHOT-12 and HAGOS) aimed to capture various dimensions of hip pain, including symptoms, daily activities, sports participation, and overall quality of life. Additionally, any changes in the patients’ regular pain medication were recorded to isolate the effect of the Botox treatment.

### Pharmacological safety considerations

The lethal dose of Botox for an adult is stated to be 40 IU/kg. (Thus, 2800 IU if weighing 70 kg.) We used 100 IU for injection into the hip joint. Side effects previously described include pain and discomfort at the injection site, as well as fever and flu-like symptoms. In very rare cases, pronounced muscle weakness, dysphagia, constipation, and aspiration pneumonia due to toxin spread may occur.

Side effects for Botox type A toxin are reversible and usually dose dependent. There have been rare post-marketing reports of adverse events, including those involving the cardiovascular system (arrhythmia and myocardial infarction), respiratory depression, and peripheral neuropathy. Serum sickness-like reactions and anaphylaxis have also been observed. Congenital malformations have been described in animals but have never been described in humans [17].

### Ethics

The study was approved by the Ethical Committee Region Midt under Project ID 1-10-72-151-19 and notified at the Danish Medicines Agency under EudraCT 2019-004488-33. It was also notified at the Data Protection Authority for permission to store and process the collected data—Legal office Region Midt Case No. 1-16-02-256-19. The study fulfilled the requirements for the conduct of clinical trials in the European Union and was monitored by Good Clinical Practice.

## Results

Eleven patients were included in the study. One patient left the study and did not receive medicine. Ten patients completed the trial.

All included patients were Caucasian women. The mean age was 37.3 years.

**Figure 3.**
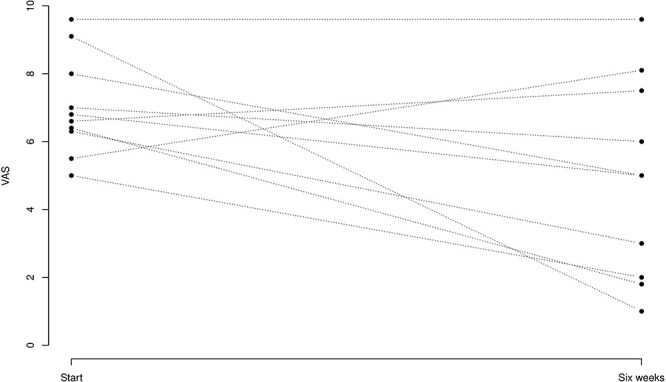
*X*-axis: VAS pain scores were 0 with no pain and 10 with the worst possible pain. *Y*-axis: start is Day 0 when the patient received the medicine in the hip joint. Six  weeks: when patients were evaluated for the second time for the VAS pain score.

A clinically significant decrease in the VAS score was observed at the 6-week control [2.13 cm (−0.03, 4.29); *P* = .0527] (Fig. 3, Table 1).

**Table 1.** Median values of the VAS pain score at the start value and after 1, 2, and 6 weeks.

	Start value before Botox injection	One week after Botox injection	Two weeks after Botox injection	Six weeks after Botox injection
VAS median	7.03 (IQR = 1.7)	6.58 (IQR = 3.0)	5.96 (IQR = 3.3)	4.90 (IQR = 5.5)

**Figure 4.**
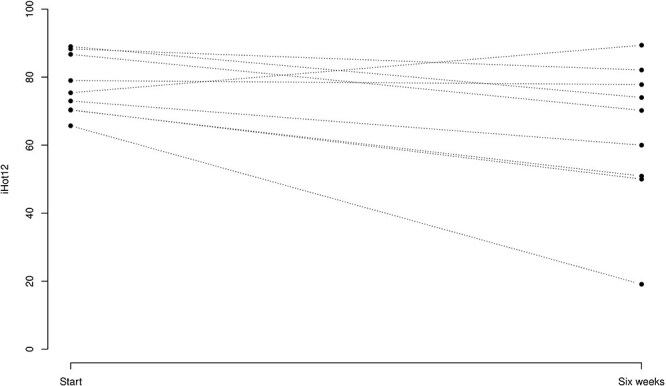
*X*-axis: iHOT-12, where patients evaluated questionnaire are transformed to a number between 0 and 100. *Y*-axis: Start is Day 0 when the patient received the medicine in the hip joint. Six weeks: when patients were evaluated for the second time with iHOT-12.

Evaluation of patient symptoms using iHOT-12 questions at the 6-week control compared to the start value showed a statistically significant decrease in symptoms [14.1 (3.01, 25.2); *P* = .0183] (Fig. 4).

**Figure 5.**
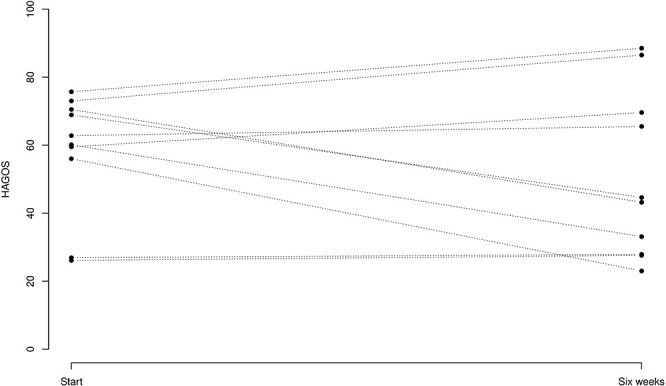
*X*-axis: HAGOS, where patients evaluated questionnaire are transformed to a number between 0 and 100. *Y*-axis: start is Day 0 when the patient received the medicine in the hip joint. Six weeks: when patients were evaluated for the second time with HAGOS.

Evaluation of patient symptoms using HAGOS questionaries at the 6-week control compared to the start value showed a nonsignificant decrease [7.0 (−6.3, 20), *P* = .2651] (Fig. 5).

Six patients had a >1.5 cm pain reduction on the VAS score. One patient had no effect on the VAS score and three patients had a raise in the VAS score.

No changes in regular pain medication were observed in any patient during the 6-week observation period.

No patients reported any side effects.

One patient in whom the medicine was caught in the joint capsule and did not spread intra-articularly reported no effect of the medicine either on the VAS score or on HAGOS or iHOT-12 (Table 2).

**Table 2.** Angle measurements before and after surgical correction: evaluation of additional surgery and complications after periacetabular osteotomy in the study group.

Surgical correction, additional surgery, and complications after periacetabular osteotomy in the study group				
	Preoperative		Postoperative	
Centre edge index side, angle measurement in degrees	Mean	17.1	Mean	32.9
Tönnis acetabular roof angle in degrees, index side	Mean	13.4	Mean	4.4
Retroversion (crossover sign in combination with posterior wall sign), number of patients		3		0
Arthrosis on X-ray		0		0
			LFCN (Lateral Femural Cutaneous Nerve)	6
			Pulmonary embolism/deep venous thrombosis	0
			Nonunion of osteotomy	0
			Motor nerve	0
			Hip arthroscopy	6
			Screw removal	7
			Total hip arthroplasty	1
			Additional surgery	0

## Discussion

This study aimed to evaluate the efficacy of Botox injections combined with lidocaine as a treatment for persistent hip pain PAO in a cohort of 10 female patients. The significant decrease in pain intensity, as measured by the VAS at a 6-week follow-up, and a statistically significant decrease in iHOT score underline Botox’s potential as a viable alternative to traditional corticosteroid treatments, which are often associated with undesirable side effects.

The results indicate that Botox injections safely can provide long-lasting pain relief for the majority of patients, which could significantly impact patient management strategies, especially for those suffering from chronic hip pain with limited treatment options. The ability of Botox to offer extended pain relief may also reduce the need for frequent medication adjustments, thus enhancing patient compliance and satisfaction. Botox might even have a longer effect than corticosteroids [18].

However, the variability in response—particularly the increased pain scores in three patients—suggests a complex interaction between the treatment and individual patient pathologies. This variability could be due to factors such as the exact placement of the injection, individual differences in Botox absorption, or underlying conditions not fully addressed by the treatment. For diagnostic purposes, the effect of Botox injections in the hip joint was found to be uncertain and less effective compared to current diagnostic tests using local anaesthetic.

Moreover, the study highlighted the critical importance of precise administration. The case where Botox was not effectively spread within the joint capsule, resulting in no pain relief, underscores the need for improved imaging techniques or guidance systems during injection to ensure optimal delivery of the treatment substance.

It is encouraging to note that no side effects were reported in this small cohort, suggesting that Botox, when used in the controlled dosage of 100 IU diluted in 3 ml NaCl solution mixed with lidocaine, offers a safer profile for patients undergoing treatment for hip pain. This safety profile, coupled with the evidence of pain reduction similar to that found in studies of the knee and shoulder, indicates that the treatment could be suitable for outpatient settings. It could have been time effective to combine the local anaesthetic test of the hip joint and the Botox test in one. At least 8 ml of lidocaine 10 mg/ml combined with 100 IE Botox diluted in 3 ml NaCl. Patient information about pain during the first 3 h post-injection separated from the following effect is important and can be helped with a score sheet for pain registration before and after 3 h after medicine injection.

One notable case was a patient who had previously experienced menstrual disorders after steroid injections in the hip and found substantial pain relief after Botox; this patient has since received repeated injections, each time with pain relief, highlighting Botox’s potential for repeated use without diminishing efficacy.

### Limitations

The study is a pilot study with only a small number of patients. The results come from comparing pain measurements before and after treatment, but the study could have been stronger with blinding and a placebo control group. The patients were followed for 6 weeks, but more extensive testing and a longer follow-up might have been beneficial to find out how long-time Botox actually reduces pain. Due to the small number, it is also difficult to give a precise idea of the variability in injection efficacy. A study with a larger number of patients might have helped to exclude inhomogeneity of sample and other potential biases nevertheless the cohort has an inherent inhomogeneity and not all the patients will have intra-articular pain and therefore a positive effect of the injection. Botox comes with a larger price than using lidocaine alone or lidocaine mixed with corticosteroids and this might be a limitation in itself for more widespread use if its effect is not significantly better.

## Conclusion

For diagnostic use of Botox in the hip joint, it seems that the pain-reducing effect is not effective enough to replace local anaesthetics. However, Botox has a promising and significant pain-reducing effect on the hip joint in the majority of patients comparable to the effects seen in knee and shoulder joints. We recommend larger-scale studies necessary to confirm these results and optimize the treatment protocol. Hip joint injection of a controlled dosage of 100 IU diluted in 3 ml NaCl solution mixed with lidocaine had no side effects in this study with a limited group of patients.

## Data Availability

All study data are stored in REDCap. Anonyminized data are safely stored locally by the corresponding author.
